# A neural modeling approach to study mechanisms underlying the heterogeneity of visual spatial frequency sensitivity in schizophrenia

**DOI:** 10.1038/s41537-024-00480-2

**Published:** 2024-07-16

**Authors:** Caroline Dugan, Basilis Zikopoulos, Arash Yazdanbakhsh

**Affiliations:** 1 Program in Neuroscience, Boston University, Boston, MA USA; 2 Human Systems Neuroscience Laboratory, Department of Health Sciences, Boston University, Boston, MA USA; 3 Department of Anatomy & Neurobiology, Boston University School of Medicine, Boston, MA USA; 4 Center for Systems Neuroscience, Boston University, Boston, MA USA; 5 Graduate Program for Neuroscience, Boston University, Boston, MA USA; 6 Computational Neuroscience and Vision Laboratory, Department of Psychological and Brain Sciences, Boston University, Boston, MA USA

**Keywords:** Neural circuits, Neuroscience

## Abstract

Patients with schizophrenia exhibit abnormalities in spatial frequency sensitivity, and it is believed that these abnormalities indicate more widespread dysfunction and dysregulation of bottom-up processing. The early visual system, including the first-order Lateral Geniculate Nucleus of the thalamus (LGN) and the primary visual cortex (V1), are key contributors to spatial frequency sensitivity. Medicated and unmedicated patients with schizophrenia exhibit contrasting changes in spatial frequency sensitivity, thus making it a useful probe for examining potential effects of the disorder and antipsychotic medications in neural processing. We constructed a parameterized, rate-based neural model of on-center/off-surround neurons in the early visual system to investigate the impacts of changes to the excitatory and inhibitory receptive field subfields. By incorporating changes in both the excitatory and inhibitory subfields that are associated with pathophysiological findings in schizophrenia, the model successfully replicated perceptual data from behavioral/functional studies involving medicated and unmedicated patients. Among several plausible mechanisms, our results highlight the dampening of excitation and/or increase in the spread and strength of the inhibitory subfield in medicated patients and the contrasting decreased spread and strength of inhibition in unmedicated patients. Given that the model was successful at replicating results from perceptual data under a variety of conditions, these elements of the receptive field may be useful markers for the imbalances seen in patients with schizophrenia.

## Introduction

**Fig. 1:**
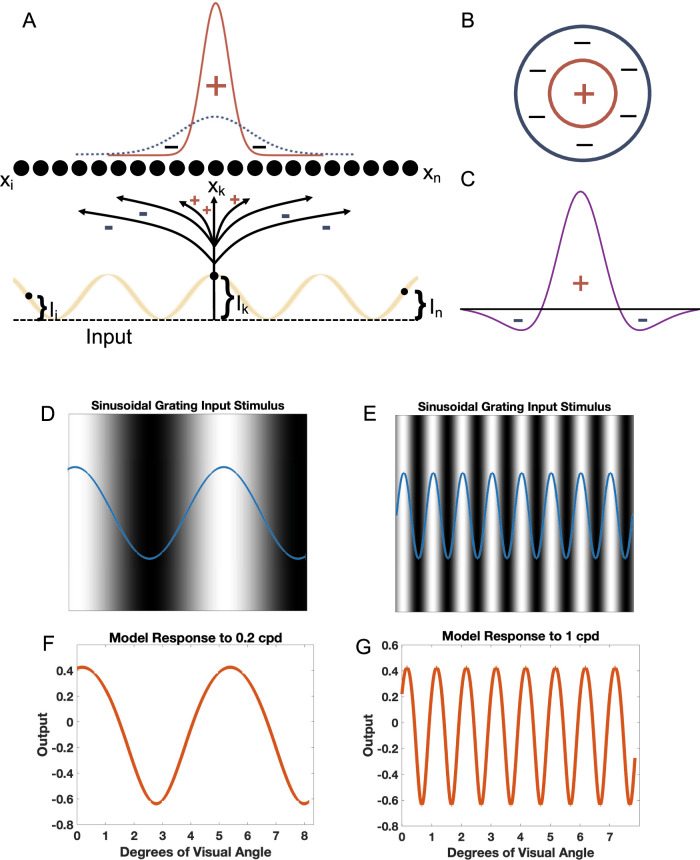
Model network, receptive field model, and sinusoidal grating input stimulus generation. Array of model neurons receiving input through excitatory and inhibitory connections. The studied inputs are luminance sin modulation (**A**). Considering an on-center/off-surround organization of receptive fields (**B**), in (**D**), we implemented excitatory and inhibitory connectivities following weighted subtraction of Gaussians (Difference of Gaussians, DoG) to approximate excitatory and inhibitory subfields of receptive field (**C**). Sinusoidal grating inputs of 0.2 (**D**) and 1 (**E**) cpd were generated. The blue sin waves represent the input stimulus while the black and white images represent the corresponding luminance gratings they represent, with black areas corresponding to minimum luminance values (trough of sin graph) and white areas corresponding to maximum luminance values (peak of sin graph). The model response to inputs of 0.2 (**F**) and 1 (**G**) cpd were then measured.

Schizophrenia is characterized by dysfunction in sensory processing that alters sensory perception, but is distinct from hallucinations^1^. Spatial frequency sensitivity, which in the visual domain describes sensitivity to patterns composed of alternating light and dark bars in a given unit of space, typically in one degree of visual angle, is a central process that is affected in schizophrenia (reviewed in ref. ^2^). Spatial frequency is expressed as cycles per degree of sine-wave gratings^3^. In other words, spatial frequency sensitivity refers to an individual’s ability to detect light and dark contrast (contrast sensitivity) when adjacent light and dark regions alternate at different frequencies in a given unit of space. At low spatial frequencies (Fig. 1D), the stimulus alternates between black and white less frequently, whereas at higher spatial frequencies (Fig. 1E), the stimulus alternates between black and white much more frequently. While spatial frequency sensitivity varies to a certain extent between individuals, people tend to be best at detecting light-dark contrast at intermediate spatial frequencies. A variety of factors can affect one’s spatial frequency sensitivity, and abnormal spatial frequency sensitivity patterns have been identified in both medicated and unmedicated patients with schizophrenia.

Medicated patients with schizophrenia who are taking either typical or atypical antipsychotics tend to exhibit decreased contrast sensitivity, although there is conflicting data regarding the range of spatial frequencies at which deficits are observed^3–10^. Previous studies have demonstrated that medicated patients can exhibit decreased sensitivity across all spatial frequencies^4,9,10^ but in some cases only at low^3,5–9^ or only at medium to high spatial frequencies^4,10^ (Table 1). Factors such as illness duration, medication type, stimulus used, and symptom type have been demonstrated to affect spatial frequency sensitivity, but a general trend of decreased sensitivity in medicated patients is consistent across studies, and it appears that this trend may be more pronounced with increased illness duration. Studies indicate that patients with an illness duration greater than ten years have decreased contrast sensitivity at all spatial frequencies, whereas medicated patients with an illness duration less than ten years exhibit decreased sensitivity at only low spatial frequencies^9^. With regards to medication type, patients taking atypical antipsychotics may experience less pronounced deficits in contrast sensitivity compared with patients taking typical antipsychotics^6,9^.

**Table 1:** Relevant findings from previous studies of spatial frequency sensitivity in patients with schizophrenia that we replicated with the single-layer feedforward visual spatial frequency sensitivity model.

Reference	Major findings	Spatial frequency sensitivity shift	Medication status	Average age and illness duration
Butler et al. 2005^3^	Deficits at low and medium spatial frequencies (0.5-7 cpd)	Selective shift downward from 0.5 to 7 cpd	Medicated	Age: 37.1 ± 1.7 years. Illness duration: 14.5 ± 1.7 years
Cimmer et al. 2006^8^	SZ patients demonstrated decreased sensitivity at 0.5 cpd	Shift downward at 0.5 cpd	Medicated	Non-deficit schizophrenia: Age: 36.8 ± 12.7 Age of onset: 26.2 ± 8.1. Deficit schizophrenia: Age: 35.7 ± 11.6 Age of onset: 25.3 ± 9.9.
Shoshina and Shelepin, 2015^9^	SZ patients <10 years: decreased sensitivity only at low spatial frequencies SZ patients >10 years: decreased sensitivity at all spatial frequencies	SZ < 10 years: downward shift at low spatial frequencies SZ > 10 years: overall downward shift of the curve	Medicated	Mean duration of illness in group 1 (less than 10 years): 3.9 ± 2.3 years Mean duration of illness in group 2 (more than 10 years): 17.3 ± 6.9 years
Butler et al. 2009^7^	Impaired contrast sensitivity at 0.5 cpd, but not at 7 and 21 cpd	Downward shift around 0.5 cpd	Medicated	Age: 36.4 ± 2.2 years Illness duration: 16.1 ± 2.0 years
Kéri et al. 2002^10^	Static stimulus: Deficits at medium and high spatial frequencies (2.9–14.4 cpd) Dynamic stimulus: Deficits across all spatial frequencies (0.5–14.4 cpd)	Static: Downward shift at medium and high spatial frequencies Dynamic: Downward shift across all spatial frequencies	Medicated	Age: 36.1 ± 7.1 years Duration of illness: 5.2 ± 1.7 years
Slaghuis, 1998^4^	Static stimulus: Negative-symptom patients: Deficits across all spatial frequencies Positive-symptom patients: deficits at medium and high spatial frequencies Drifting condition: Negative-symptom patients: Deficits across all spatial frequencies Positive-symptom patients: decreased sensitivity at medium and high spatial frequencies	Negative-symptom patients: Downward shift across spatial frequencies (Static and drifting) Positive-symptom patients: Downward shift at medium and high spatial frequencies (Static and drifting)	Medicated	Age, positive symptoms group: 30.1 ± 4.7 Age, negative symptoms group: 30 ± 4.5 Duration of illness, positive symptoms group: 7.2 ± 5.2 Duration of illness, negative symptoms group: 8.2 ± 8.2
Kiss et al. 2010^11^	Magnocellular-biased trials: Increased sensitivity at low spatial frequencies Parvocellular-biased trials: No significant change	Magnocellular-biased stimuli: Upward shift from 0.25 to 4 cpd	Unmedicated	Patients were never medicated, first-episode Mean age: 25.7 ± 8.2
Kelemen et al. 2013^12^	Magnocellular-biased trials: Increased sensitivity at low spatial frequencies when unmedicated Parvocellular-biased trials: No significant change when unmedicated	Magnocellular-biased: Upward shift at low spatial frequencies	Participants unmedicated at time of study	Mean duration untreated psychosis: 9.7 ± 6.1 months”
Cadenhead et al. 2013^5^	Achromatic stimuli: Unmedicated patients demonstrated higher sensitivity, whereas medicated patients demonstrated lower contrast sensitivity at 1.22 cpd Chromatic stimuli: Unmedicated and medicated patients demonstrated decreased sensitivity at 1.22 cpd	Unmedicated: slight upward shift of luminance contrast sensitivity curve around 1.22 cpd Medicated: downward shift of luminance contrast sensitivity curve around 1.22 cpd	Medicated and unmedicated patients	Mean age: 35.7 ± 11.3
Chen et al. 2003^6^	Atypical antipsychotics: Unimpaired contrast sensitivity Typical antipsychotics: Decreased contrast sensitivity Unmedicated patients: Increased contrast sensitivity	Atypical: no change Typical: shift downward at tested 0.5 cpd Unmedicated: shift upward at 0.5 cpd	Patients taking atypical, typical, and no antipsychotics were studied	Average duration of illness: 16.0 ± 9.2 years

*SZ* Schizophrenia

Despite challenges in research involving unmedicated patients with schizophrenia, studies have identified that, in general, unmedicated patients tend to exhibit increased contrast sensitivity. Some observed this increase at low spatial frequencies^5,6,11,12^, though the trend of increased spatial frequency may be generalized across the range of spatial frequencies (Table 1). The spatial frequency range at which abnormalities are observed may be dependent on illness stage, as first-episode, medication-naïve patients have been shown to exhibit increased sensitivity at low spatial frequencies^11^.

Thus, expected contrast sensitivity patterns for medicated and unmedicated patients would follow opposite trends, with decreased sensitivity in the low, medium-high, or all spatial frequency ranges for the first group and increased sensitivity in the low or all spatial frequency ranges for the latter group. Studies provide clues suggesting that disruptions in three major neurotransmitters, glutamate, dopamine, and GABA, contribute, in tandem, to an imbalance of excitation and inhibition in the brains of those with schizophrenia^13–27^. This balance can be further altered when factors such as medication and illness duration are considered, and it is hypothesized that this alteration in the balance of excitation and inhibition contributes to the visual processing abnormalities observed in medicated and unmedicated patients with schizophrenia.

In addition to visual perception changes and hallucinations, visual processing abnormalities have been linked to several other aspects of schizophrenia. Certain visual changes in at-risk individuals have been associated with later development of schizophrenia, and visual impairments are correlated with reduced real-world functioning in patients^28–31^. Additionally, low-level visual processing abnormalities in schizophrenia, such as altered spatial frequency sensitivity, may indicate more widespread dysfunction, suggesting that there is a bottom-up component of the disorder^32^. Growing evidence suggests that deficits in sensory processing in individuals with schizophrenia can provide clues into the disorder’s overall mechanism. However, even though the visual system provides distinct advantages for the study of sensory processes^33^, the involvement of visual circuit components and mechanisms in the pathophysiology of schizophrenia is not well understood. To address this gap, we developed a rate-based feedforward model to test the overall impact of hypothesized changes in bottom-up visual excitatory networks that take into account local inhibitory circuit modulation. Using this approach, we modulated the modeled excitatory and inhibitory receptive field subfields and translated all receptive field changes to deviations in the balance of excitation and inhibition. From there, we were able to infer the perceptual outcomes linking our model output with previous data collected in perceptual studies. Finally, we investigated the model’s performance to illustrate how sensory processing abnormalities in schizophrenia connect perceptual deficits with potential underlying excitatory and inhibitory changes in medicated and unmedicated individuals with schizophrenia.

## Materials and methods

We developed a rate-based neural model of a primary visual circuit that can simulate bottom-up processing of pairs of interconnected areas in the central visual pathway, including (a) retinal input to the lateral geniculate nucleus (LGN) of the thalamus, the first-order visual thalamic nucleus that relays visual input to the cortex, (b) from LGN to the primary visual cortex (area 17), and (c) feedforward corticocortical connections between visual association areas^34–42^. The parameterized, rate-based, feedforward neural model (Eq. (1)), in which parameters point to the extent and amplitude of excitation and inhibition and the rate indicates the activity of neurons, receives an input array, and through its connections excites and inhibits the 4000 model neurons. The connectivity follows an on-center/off-surround organization^43–52^. The current model size allows for sampling of visual inputs with the appropriate resolution. Receptive field organization was modeled using a Difference of Gaussians (DoG) method. Model neurons were then presented with sinusoidal grating input with varying spatial frequencies, and the model’s response was measured to generate contrast sensitivity curves (Fig. 1A). Following a broad range of single or combined parameter changes, described in detail below, we compared model responses with key behavioral/perceptual findings from previous studies of spatial frequency sensitivity in patients with schizophrenia (Table 1), with the aim to identify possible circuit mechanisms and changes at the receptor or neurotransmitter levels at multiple states of the disorder.

### Difference of Gaussians

The Difference of Gaussians (DoG) provides an on-center/off-surround parametrizable method for the model receptive fields. The Gaussians for the excitatory on-center and inhibitory off-surround are concentric and the difference between the two approximates an on-center/off-surround receptive field organization. With the model feedforward architecture from the input array to the model neuron array following on-center/off-surround interaction, we measured the model’s response to visual stimuli with varying spatial frequency.

### Neural Activity

The activity of each neuron in the model, represented by vector **x** (Fig. 1A), is determined by the following shunting equation^47,48,53,54^:

1 $$\frac{d{\bf{x}}}{{dt}}=-A{\bf{x}}+\left(B-{\bf{x}}\right){{\bf{I}}}_{{\bf{ex}}}-\left(C+{\bf{x}}\right)\,{{\bf{I}}}_{{\bf{inh}}}$$

Constants *A*, *B*, and *C* represent the neural activity decay term, neural activity upper limit, and neural activity lower limit, respectively. Bold variables represent arrays. Model neurons array activity (**x**) was dependent on the excitatory (**I**_**ex**_) and the inhibitory (**I**_**inh**_) components of the input array: **I**_**ex**_ refers to the input array (**I**) convolved with the excitatory Gaussian (**G**_**ex**_), and **I**_**inh**_ refers to the input array (**I**) convolved (*) with the inhibitory Gaussian (**G**_**inh**_):

2 $${{\bf{I}}}_{{\bf{ex}}}={\bf{I}}* {{\bf{G}}}_{{\bf{ex}}}$$

3 $${{\bf{I}}}_{{\bf{inh}}}={\bf{I}}* {{\bf{G}}}_{{\bf{inh}}}$$

**G**_**ex**_ and **G**_**inh**_ follow the equations:

4 $${\bf{G}}_{{\bf{ex}}}={{Amp}}\,_{{ex}}\,{e}^{(-\frac{{{\bf{x}}}^{2}}{{2\sigma}\,_{{ex}}^{2}})}$$

5 $${\bf{G}}_{{\bf{inh}}}={{Amp}}\,_{{inh}}\,{e}^{(-\frac{{{\bf{x}}}^{2}}{{2\sigma}\,_{{inh}}^{2}})}$$

In which **x** ranges from −5 *σ*_*ex/inh*_ to 5 *σ*_*ex/inh*_. All parameters and symbols are summarized in the abbreviation list at the end of this section along with their values in Supplementary Table 1. *Amp*_*ex*_ and *Amp*_*inh*_ refer to the amplitude or height of the excitatory and inhibitory subfields (Gaussians), respectively. *σ*_*ex*_ and *σ*_*inh*_ refer to the width of the excitatory and inhibitory subfields (Gaussians), respectively.

Our current neural modeling results are based on the equilibrium state of neural dynamics of Eq. (1):

6 $${{\bf{x}}}_{{\bf{eq}}}=\frac{B{{\bf{I}}}_{{\bf{ex}}}-C{{\bf{I}}}_{{\bf{inh}}}}{A+{{\bf{I}}}_{{\bf{ex}}}+{{\bf{I}}}_{{\bf{inh}}}}$$

where **x**_**eq**_ is model neurons array activity at equilibrium.

Substituting **I**_**ex**_ and **I**_**inh**_ values from Eqs. (2)–(3) yields:

7 $${{\bf{x}}}_{{\bf{eq}}}=\frac{{\bf{I}}* {(B{\bf{G}}}_{{\bf{ex}}}-C{{\bf{G}}}_{{\bf{inh}}})}{A+{\bf{I}}* ({{\bf{G}}}_{{\bf{ex}}}+{{\bf{G}}}_{{\bf{inh}}})}$$

The equilibrium of Eq. 7 shows the normalized by input weighted subtraction of excitatory and inhibitory Gaussian or Difference of Gaussians (DoG) as the effective combined excitatory and inhibitory subfields processing the model inputs.

Considering DoG, we set the inhibitory Gaussian, with a larger *σ* (*σ*_*inh*_) and smaller amplitude (*Amp*_*inh*_), and the excitatory Gaussian, with a smaller sigma (*σ*_*ex*_) and a larger amplitude (*Amp*_*ex*_) for all of the reported results. The result of such Gaussians’ subtraction mimics an on-center/off-surround receptive field^55^, given that it has a positive center flanked by two negative, inhibitory regions (Fig. 1B, C). Marr and Hildreth^55^ showed that the ratio of *σ*_*inh*_ to *σ*_*ex*_ for optimal contrast registration is 1.6:1, which we implemented for the base state of simulations and by varying *σ*s, amplitudes, and combinations, we examined their impacts on the neural model spatial frequency sensitivity.

### Input generation

To determine the model’s response to varying spatial frequencies, sinusoidal inputs with varying spatial frequencies were generated and used (Eq. 8). These inputs represent luminance gratings with varying spatial frequencies (Fig. 1D, E). The peaks of the sinusoidal gratings correspond to areas of maximum luminance, while the troughs of the sinusoidal gratings correspond to areas of minimum luminance.

8 $$y=a\,{\mathrm{sin}}(2{\rm{\pi}}f\,i)$$

*a* refers to the amplitude of the sinusoidal grating, which was held constant at 0.1 across model trials. *f* refers to spatial frequency used, which ranged from 0.1 to 100 cycles per degree (cpd). Position is represented by the variable *i*. The model was shown 200 sinusoidal gratings with spatial frequencies ranging from 0.1 to 100 cpd and its response to each grating was measured and plotted to create the contrast sensitivity curves.

### Parameter selection

Across model trials, amplitude and *σ* values were altered while values of *A*, *B*, and *C* were kept constant. *A*, *B*, and *C* were set to values of 1, 10.1, and 5. Parameter selections for each model run are reflected in Supplementary Table 1, and the following abbreviation list reflects a summary of the parameters and symbols used in equations.

*Abbreviation list reflecting the parameters and symbols used in equations*

**Table:** 

*Amp*_*ex*_	Height of the excitatory Gaussian
*Amp*_*inh*_	Height of the inhibitory Gaussian
*σ*_*ex*_	Width of the excitatory Gaussian
*σ*_*inh*_	Width of the inhibitory Gaussian
*	Convolution
*A*	Neural activity decay term
*B*	Neural activity upper limit
*C*	Neural activity lower limit
*a*	Sinusoidal amplitude
*f*	Spatial frequency
*i*	Sinusoidal position
θ	Cosine similarity angle between sensitivity curves

### Measuring model response

To determine the model’s contrast sensitivity for varying spatial frequencies, the minimum and maximum values of the model neurons array response were determined for each spatial frequency. Given the model’s connectivity, architecture, and the input stimuli used, the model neuron array has maximum activities at maximum luminance, or sinusoidal peak locations, and the minimum activities are at minimum luminance, or sinusoidal trough locations (Fig. 1F, G). We therefore consider the contrast readout of the model based on maximum and minimum activities for each sin stimulus as the model representation of contrast sensitivity:

9 $${Contrast\; Sensitivity}={Response\; Peak}-{Response\; Trough}$$

In other words, for each sinusoidal grating input, the model’s representation of contrast sensitivity was determined by subtracting the neuron array’s minimum activity from the maximum activity. Correspondingly, in perceptual studies (e.g., see Table 1), spatial frequency sensitivity is determined by the inverse of observers’ contrast detection threshold at various spatial frequencies as a pointer to the magnitude of neural representation of contrast.

### Spatial frequency ranges

By comparing model response patterns with existing data from perceptual studies in patients with schizophrenia, the low, medium, and high spatial frequency ranges were determined^3,7,9,11^. Low spatial frequency ranged between 0.1-4 cpd, medium spatial frequency between 4-10 cpd, and high spatial frequency between 10-25 cpd. We considered consistent ranges for low, mid, and high spatial frequencies, however, the exact ranges in prior studies could vary depending on upper and lower limits for low, medium, and high spatial frequencies as well as the stimulus type (i.e., Gabor vs. uniform spatial frequency). Therefore, certain reported differences from perceptual studies at medium spatial frequencies, for example, may slide into our uniformly defined low spatial frequency range; hence, studies reported in Table 1 should be considered with their uniquely defined spatial frequency ranges.

### Analysis

The model contrast sensitivity is represented with a vector. Cosine similarity (cosSim) based angle (θ) between the model sensitivity vector (MSV) in a given condition and the overall model “base” contrast sensitivity vector (MBCSV) could be calculated using Eq. 10 ($\bullet$ stands for dot product) and 11:

10 $$\cos {Sim}=\frac{{MSV}\,{{\bullet }}\,{MBCSV}}{\left|{MSV}\right|{\rm{|}}{MBCSV}{\rm{|}}}$$

11 $${\rm{\theta }}={\rm{arcCos}}\left(\cos {\rm{Sim}}\right)$$

The normalized difference index (NDI) between the MSV and MBCSV at low, medium, and high spatial frequencies was determined according to Eq. 12:

12 $${NDI}=\frac{\left|{MSV}\right|-{\rm{|}}{MBCSV}{\rm{|}}}{\left|{MSV}\right|+{\rm{|}}{MBCSV}{\rm{|}}}$$

in which |…| represents the vector size, and the NDI significance was determined using t-test.

## Results

By measuring the neural model contrast sensitivity with different excitatory and inhibitory parameters and comparing the model contrast sensitivity with each parameter set with contrast sensitivity perceptual data from medicated and unmedicated patients with schizophrenia as well as healthy controls, we could approximate and compare the excitation / inhibition balance in each group. Supplementary Table 1 summarizes the model’s parameter set modifications from base set (control) that result in matched contrast sensitivity to medicated and unmedicated patients, thus providing a guide to the results of each parameter modification. Table 2 summarizes the model’s parameter set modifications from base set (control) that result in contrast sensitivity changes and the best fit replication that was determined by examining the NDI value and magnitude for each model sensitivity curve at low, medium, and high spatial frequencies as well as the overall curve shape, as reported by cosSim and θ in low, medium, and high spatial frequency ranges (see also Supplementary Table 2).

**Table 2:** Model excitatory and inhibitory subfield changes matching patient perceptual contrast sensitivity data.

Patient type	Replicated results	Needed model modifications			Best fit replication
Medicated	Decreased sensitivity at low spatial frequencies	↑ *σ*_*ex*_ ↓ *Amp*_*ex*_ ↓ *σ*_*ex*_ & ↓ *Amp*_*ex*_ ↑ *σ*_*ex*_ & ↓ *Amp*_*ex*_	↓ *σ*_*inh*_ ↑ *Amp*_*inh*_ ↑ *σ*_*inh*_ & ↑ *Amp*_*inh*_ ↓ *σ*_*inh*_ & ↑ *Amp*_*inh*_	↓ *σ*_*ex*_ & ↓ *σ*_*inh*_ ↑ *σ*_*ex*_ & ↑ *σ*_*inh*_ ↓ *Amp*_*ex*_ & ↓ *Amp*_*inh*_	↑ *σ*_*inh*_ & ↑ *Amp*_*inh*_
	Decreased sensitivity at medium & high spatial frequencies	↑ *σ*_*ex*_ ↓ *Amp*_*ex*_ ↑ *σ*_*ex*_ & ↑ *Amp*_*ex*_	↑ *σ*_*ex*_ & ↓ *Amp*_*ex*_ ↓ *σ*_*inh*_ ↑ *Amp*_*inh*_	↓ *σ*_*inh*_ & ↑ *Amp*_*inh*_ ↑ *σ*_*ex*_ & ↑ *σ*_*inh*_ ↓ *Amp*_*ex*_ & ↓ *Amp*_*inh*_	↑ *σ*_*ex*_ & ↑ *σ*_*inh*_
	Overall decreased sensitivity	↑ *σ*_*ex*_ ↓ *Amp*_*ex*_ ↑ *σ*_*ex*_ & ↓ *Amp*_*ex*_	↓ *σ*_*inh*_ ↑ *Amp*_*inh*_ ↓ *σ*_*inh*_ & ↑ *Amp*_*inh*_	↑ *σ*_*ex*_ & ↑ *σ*_*inh*_ ↓ *Amp*_*ex*_ & ↓ *Amp*_*inh*_	↓ *Amp*_*ex*_ & ↓ *Amp*_*inh*_
Unmedicated	Increased sensitivity at low spatial frequencies	↓ *σ*_*ex*_ ↑ *Amp*_*ex*_ ↑ *σ*_*ex*_ & ↑ *Amp*_*ex*_	↓ *σ*_*ex*_ & ↑ *Amp*_*ex*_ ↑ *σ*_*inh*_ ↓ *Amp*_*inh*_	↓ *σ*_*inh*_ & ↓ *Amp*_*inh*_ ↑ *σ*_*inh*_ & ↓ *Amp*_*inh*_	↓ *σ*_*inh*_ & ↓ *Amp*_*inh*_
	Overall increased sensitivity	↓ *σ*_*ex*_ ↑ *Amp*_*ex*_ ↓ *σ*_*ex*_ & ↑ *Amp*_*ex*_	↑ *σ*_*inh*_ ↓ *Amp*_*inh*_ ↑ *σ*_*inh*_ & ↓ *Amp*_*inh*_	↑ *Amp*_*ex*_ & ↑ *Amp*_*inh*_	↓ *σ*_*ex*_ & ↑ *Amp*_*ex*_

Best fit replication was determined by examining the NDI value and magnitude for each model sensitivity curve at low, medium, and high spatial frequencies as well as the overall curve shape, as reported by cosSim and θ (see Supplementary Table 2).

### Effects of changes in excitation

#### Impact of changes in excitation extent (*σ*_*ex*_)

**Fig. 2:**
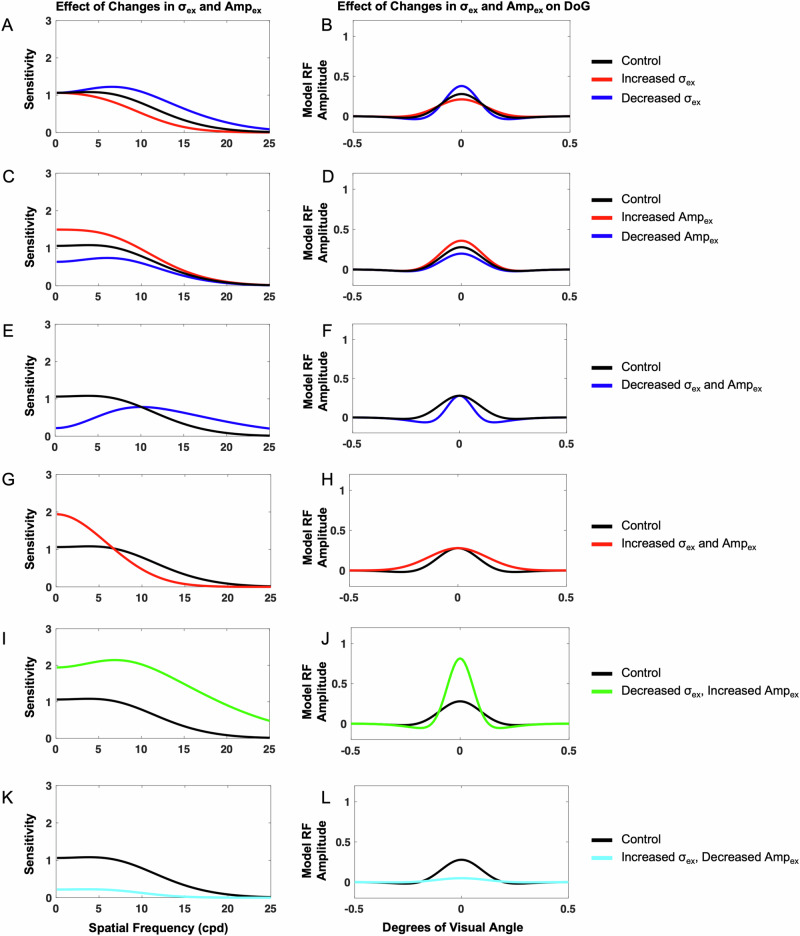
Isolated and concurrent impact of changing excitatory extent (*σ*_*ex*_) and excitatory amplitude (*Amp*_*ex*_) on the neural model spatial frequency sensitivity and receptive field profile (DoG). Impacts on spatial frequency (**A**) and DoG profile (**B**) were measured when *σ*_*ex*_ was increased from neural model base (control) to a value of 1.2 (red) and decreased to a value of 0.8 (blue). Impacts on spatial frequency (**C**) and DoG profile (**D**) were measured when *Amp*_*ex*_ was increased from neural model base (control) to a value of 1.2 (red) and decreased to a value of 0.8 (blue). Concurrent impacts of *σ*_*ex*_ and *Amp*_*ex*_ were measured when *σ*_*ex*_ and *Amp*_*ex*_ were decreased to 0.6 (**E**, **F**), when *σ*_*ex*_ and *Amp*_*ex*_ were increased to 1.4 (**G**, **H**), when *σ*_*ex*_ was decreased to 0.6 and *Amp*_*ex*_ was increased to 1.4 (**I**, **J**), and when *σ*_*ex*_ was increased to 1.4 and *Amp*_*ex*_ was decreased to 0.6 (**K**, **L**).

When the excitatory subfield extent was decreased, the model demonstrated increased sensitivity across spatial frequencies, and when the excitatory subfield extent was increased, the model demonstrated decreased sensitivity across spatial frequencies. To determine the isolated impact of altering the excitatory subfield extent (*σ*_*ex*_), we held *σ*_*inh*_, *Amp*_*inh*_, and *Amp*_*ex*_ constant at standard values of 1.6, 1, and 1, respectively. *σ*_*ex*_ was then varied (Supplementary Table 1), and the resulting contrast sensitivity vs. input spatial frequency curves were compared to the control base (Fig. 2A, B). Varying *σ*_*ex*_ from the base value of 1 resulted in an overall shift of the contrast sensitivity curve, changing both overall sensitivity and the spatial frequency at which the model was most sensitive. Decreasing *σ*_*ex*_ to a value of 0.8 resulted in increased sensitivity across spatial frequencies, and the spatial frequency at which the model demonstrated highest sensitivity was shifted rightward to higher spatial frequencies when compared with the model base sensitivity curve. Alternatively, increasing *σ*_*ex*_ to a value of 1.2 resulted in decreased sensitivity across spatial frequencies and a shift in maximum sensitivity toward lower spatial frequencies (Fig. 2A). Therefore, decreasing *σ*_*ex*_ resulted in maximum sensitivity at higher spatial frequencies and overall increased sensitivity, while increasing *σ*_*ex*_ resulted in maximum sensitivity at very low spatial frequencies and overall decreased sensitivity.

#### Impact of changes in excitation amplitude (*Amp*_*ex*_)

When the excitatory subfield amplitude was increased, the model demonstrated increased sensitivity across spatial frequencies, and when the excitatory subfield amplitude was decreased, the model demonstrated decreased sensitivity across spatial frequencies. To determine isolated impact of *Amp*_*ex*_, the three other parameters, *σ*_*ex*_, *σ*_*inh*_, and *Amp*_*inh*_ were held constant at standard values of 1, 1.6, and 1, respectively, while *Amp*_*ex*_ was varied (Fig. 2C, D). Varying *Amp*_*ex*_ from the base (control) value of 1 resulted in changes in both overall sensitivity and the spatial frequency at which maximum sensitivity occurred. Increasing *Amp*_*ex*_ to a value of 1.2 resulted in an overall increase in sensitivity across spatial frequencies, and the spatial frequency at which the model demonstrated maximum sensitivity was shifted leftward toward lower spatial frequencies when compared with the control. Increasing *Amp*_*ex*_ also resulted in a lack of a distinct peak, with maximum sensitivity occurring within a small range of spatial frequencies rather than at one distinct spatial frequency. Decreasing *Amp*_*ex*_ to 0.8 resulted in an overall decrease in sensitivity across spatial frequencies, and maximum sensitivity occurred at higher spatial frequencies when compared with the base control (*Amp*_*ex*_ = 1). It is also important to note that decreasing *Amp*_*ex*_ resulted in the appearance of a more distinct peak in the spatial frequency sensitivity curve. Thus, varying *Amp*_*ex*_ affected overall sensitivity, the spatial frequency at which maximum sensitivity occurred, and peak emergence in the sensitivity curve (Fig. 2C).

#### Impact of concurrent changes in excitatory spatial extent (*σ*_*ex*_) and strength (*Amp*_*ex*_)

When the excitatory subfield extent and amplitude were both simultaneously decreased, the model demonstrated decreased sensitivity at low spatial frequencies and increased sensitivity at higher spatial frequencies. When the excitatory subfield extent and amplitude were both simultaneously increased, the model demonstrated increased sensitivity at low spatial frequencies and decreased sensitivity at higher spatial frequencies. When the excitatory subfield extent was decreased while the excitatory subfield amplitude was increased, the model demonstrated increased sensitivity across spatial frequencies. When the excitatory subfield extent was increased while the excitatory subfield amplitude was decreased, the model demonstrated decreased sensitivity across spatial frequencies. To measure the concurrent impact of *σ*_*ex*_ and *Amp*_*ex*_, *σ*_*inh*_ and *Amp*_*inh*_ values were held constant at 1.6 and 1, respectively. *Amp*_*ex*_ and *σ*_*ex*_ were then simultaneously varied from their control values of 1 and the resulting contrast sensitivity curves were compared with the control curve (Fig. 2E–L). When *σ*_*ex*_ was decreased to 0.6 and *Amp*_*ex*_ was also decreased to 0.6, the model exhibited decreased sensitivity at low spatial frequencies and increased sensitivity at higher spatial frequencies (Fig. 2E, F). Peak sensitivity also shifted to a higher spatial frequency when compared with the control. Increasing *σ*_*ex*_ to 1.4 and *Amp*_*ex*_ to 1.4 resulted in increased sensitivity at low spatial frequencies, into the low end of the medium spatial frequency range, and decreased sensitivity at higher spatial frequencies (Fig. 2G, H). The spatial frequency at which the model was maximally sensitive was lower compared to the control, and the sensitivity curve demonstrated less of a distinct peak. When *σ*_*ex*_ was decreased to 0.6 while *Amp*_*ex*_ was simultaneously increased to 1.4, the model demonstrated increased sensitivity across spatial frequencies, and maximum sensitivity occurred at a higher spatial frequency compared to the control (Fig. 2I, J). Increasing *σ*_*ex*_ to 1.4 while decreasing *Amp*_*ex*_ to 0.6 resulted in overall decreased sensitivity across spatial frequencies, and maximum sensitivity occurred at a lower spatial frequency compared to the control (Fig. 2K–L). Overall, concurrent changing of *σ*_*ex*_ and *Amp*_*ex*_ affected the preferred spatial frequency of the model toward lower and higher spatial frequencies.

### Impacts of modified inhibition

#### Impact of changes in inhibition extent (*σ*_*inh*_)

**Fig. 3:**
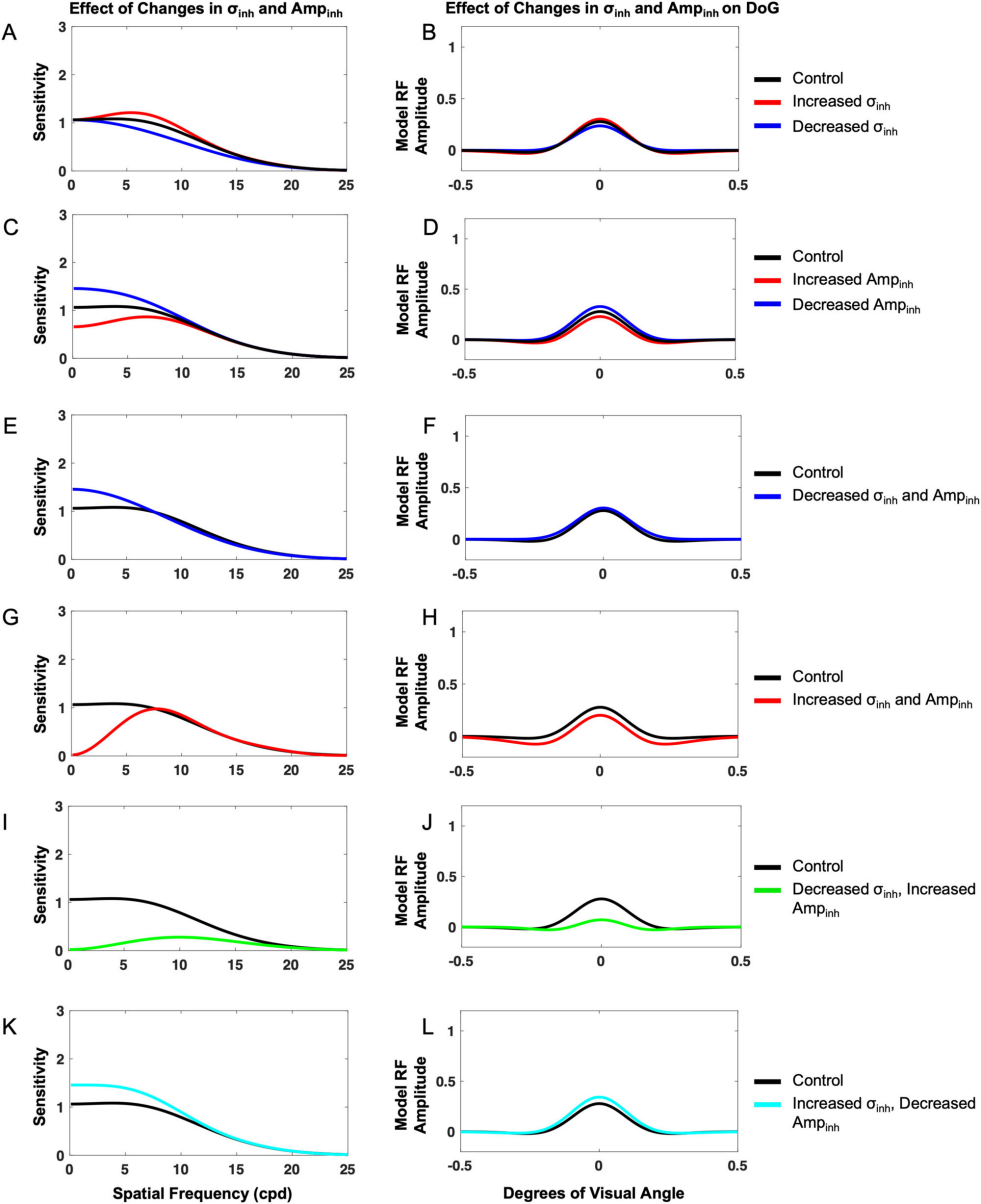
Isolated and concurrent impact of changing inhibitory extent (*σ*_*inh*_) and inhibitory amplitude (*Amp*_*inh*_) on the neural model spatial frequency sensitivity and receptive field profile (DoG). Impacts on spatial frequency (**A**) and DoG profile (**B**) were measured when *σ*_*inh*_ was increased from neural model base (control) to a value of 2.0 (red) and decreased to a value of 1.2 (blue). Impacts on spatial frequency (**C**) and DoG profile (**D**) were measured when *Amp*_*inh*_ was increased from neural model base (control) to a value of 1.4 (red) and decreased to a value of 0.6 (blue). Concurrent impacts of *σ*_*inh*_ and *Amp*_*inh*_ were measured when *σ*_*inh*_ and *Amp*_*inh*_ were decreased to values of 1.2 and 0.6. respectively (**E**, **F**), when *σ*_*inh*_ and *Amp*_*inh*_ were increased to 2.0 (**G**, **H**), when *σ*_*inh*_ was decreased to 1.2 and *Amp*_*inh*_ was increased to 2.0 (**I**, **J**), and when *σ*_*inh*_ was increased to 2.0 and *Amp*_*inh*_ was decreased to 0.6 (**K**, **L**).

When the inhibitory subfield extent was decreased, the model demonstrated decreased sensitivity across spatial frequencies, and when the inhibitory subfield extent was increased, the model demonstrated increased sensitivity across spatial frequencies. To measure the isolated impact of *σ*_*inh*_, values of *σ*_*ex*_, *Amp*_*ex*_, and *Amp*_*inh*_ were held constant at the base value of 1. Then, *σ*_*inh*_ were varied and the resulting contrast sensitivity curves were compared with the control base curve, which was generated using a *σ*_*inh*_ value of 1.6 (Fig. 3A, B). Varying *σ*_*inh*_ resulted in changes in overall sensitivity and the peak pattern in the sensitivity curve. Decreasing *σ*_*inh*_ to 1.2 resulted in overall decreased sensitivity, although these effects were more pronounced at low to medium spatial frequencies, with the lack of a distinct peak, although maximum sensitivity occurred at lower spatial frequencies when compared with the base curve. When *σ*_*inh*_ was increased to a value of 2.0, however, the model had a more distinct peak, demonstrating a stronger preference for its peak spatial frequency. The model’s spatial frequency sensitivity curve also demonstrated a slight rightward shift toward higher spatial frequencies. Additionally, the model exhibited increased sensitivity across spatial frequencies, but these effects were more pronounced at low to medium spatial frequencies.

#### Impact of changes in inhibition strength (*Amp*_*inh*_)

When the inhibitory subfield amplitude was decreased, the model demonstrated increased sensitivity across spatial frequencies, and when the inhibitory subfield amplitude was increased, the model demonstrated decreased sensitivity across spatial frequencies. To measure the isolated impact of *Amp*_*inh*_, we held *σ*_*inh*_, *σ*_*ex*_, and *Amp*_*ex*_ constant at base values of 1.6, 1, and 1, respectively, and *Amp*_*inh*_ was varied from the base value of 1, and the resulting contrast sensitivity curves were compared with the control curve, which was generated using *Amp*_*inh*_ with value of 1 (Fig. 3C, D). Changing *Amp*_*inh*_ most clearly altered the model’s sensitivity at low to medium spatial frequencies as well as the spatial frequency at which the model was maximally sensitive. Decreasing *Amp*_*inh*_ from based 1 to 0.6 (from its base 1) resulted in increased sensitivity across spatial frequencies, although these effects were most pronounced at low to medium spatial frequencies, and a shift in preference toward low spatial frequencies. The model’s response also lacked a distinct peak when *Amp*_*inh*_ was decreased. Increasing *Amp*_*inh*_ from base 1 to 1.4, however, resulted in overall decreased sensitivity, although these effects were most pronounced at low to medium spatial frequencies. Additionally, when *Amp*_*inh*_ was increased, the model was maximally sensitive at higher spatial frequencies when compared with the control, and the sensitivity curve had a more distinct peak. Therefore, *Amp*_*inh*_ had effects on the model sensitivity peak, sensitivity at low to medium spatial frequencies, and preferred spatial frequency.

#### Impact of concurrent changes in inhibition extent (*σ*_*inh*_) and amplitude (*Amp*_*inh*_)

When the inhibitory subfield extent and amplitude were both simultaneously decreased, the model most notably demonstrated increased sensitivity at low spatial frequencies. When the inhibitory subfield extent and amplitude were both simultaneously increased, the model demonstrated decreased sensitivity at low-medium spatial frequencies and increased sensitivity at high spatial frequencies. When the inhibitory subfield extent was decreased while the inhibitory subfield amplitude was increased, the model demonstrated decreased sensitivity across spatial frequencies. When the inhibitory subfield extent was increased while the inhibitory subfield amplitude was decreased, the model demonstrated increased sensitivity across spatial frequencies. To determine the impact of concurrent *σ*_*inh*_ and *Amp*_*inh*_ changes, *σ*_*ex*_ and *Amp*_*ex*_ were both held constant at the base (control) value of 1. *Amp*_*inh*_ and *σ*_*inh*_ were then simultaneously varied and the resulting curves were compared with the control sensitivity curve generated with values 1 and 1.6 for *Amp*_*inh*_ and *σ*_*inh*_, respectively (Fig. 3E–L). When *σ*_*inh*_ was decreased to 1.2 and *Amp*_*inh*_ was simultaneously decreased to 0.6, the model demonstrated increased sensitivity at low spatial frequencies, slightly increased sensitivity at medium spatial frequencies, and slightly decreased sensitivity at high spatial frequencies. The most pronounced effect was increased sensitivity at low spatial frequencies (Fig. 3E, F) The model also lacked a distinct peak in the sensitivity curve in this condition. When *σ*_*inh*_ and *Amp*_*inh*_ were both increased to 2, the model demonstrated decreased contrast sensitivity at low spatial frequencies and medium spatial frequencies and slightly increased contrast sensitivity at high spatial frequencies. The effects were most pronounced in the decreased sensitivity to low spatial frequencies (Fig. 3G, H). The model also demonstrated a more distinct peak when compared with the base control curve, and maximum sensitivity occurred at a higher spatial frequency than the control. When *σ*_*inh*_ was decreased to 1.2 and *Amp*_*inh*_ was simultaneously increased to 2, the model demonstrated overall decreased sensitivity across spatial frequencies (Fig. 3I, J). Moreover, the model demonstrated a distinct peak in its spatial frequency sensitivity curve, with maximum sensitivity occurring at higher spatial frequencies than the control condition. When *σ*_*inh*_ was increased to 2 while *Amp*_*inh*_ was decreased to 0.6, the model demonstrated overall increased contrast sensitivity (Fig. 3K–L). In this condition, the model lacked the appearance of a distinct peak in its sensitivity curve. Concurrent variation of *σ*_*inh*_ and *Amp*_*inh*_ demonstrated effects on the appearance of a distinct peak, preferred spatial frequency, and overall contrast sensitivity of the model.

### Impact of changes in receptive field size and subfields strength

#### Maintaining the ratio of excitation (*σ*_*ex*_) to inhibition (*σ*_*inh*_) subfield extents

**Fig. 4:**
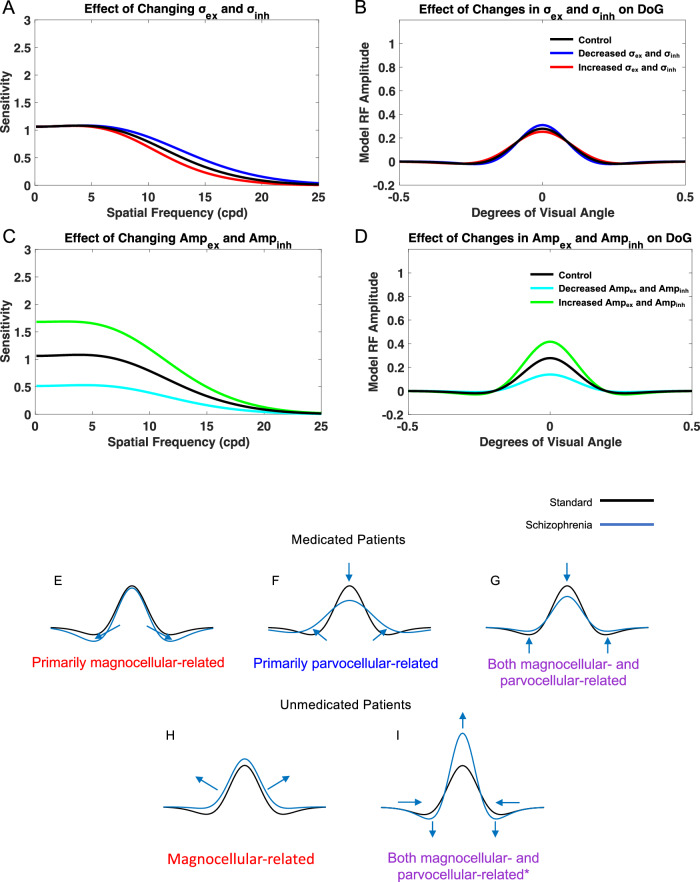
Impact of changing model receptive field size and amplitude while maintaining the ratio of excitatory to inhibitory subfields and summary of excitation/inhibition imbalance model parameter settings compared to control to replicate contrast sensitivity of medicated and unmedicated patients with schizophrenia. Spatial frequency sensitivity (**A**) and DoG (**B**); impacts were measured when *σ*_*ex*_ and *σ*_*inh*_ were altered by a factor of 0.9 (blue) and 1.1 (red). Spatial frequency sensitivity (**C**) and DoG (**D**); impacts were also measured when *Amp*_*ex*_ and *Amp*_*inh*_ were altered by a factor of 0.5 (cyan) and 1.5 (green). Based on the existing contrast sensitivity perceptual data in medicated and unmedicated patients with schizophrenia and matching the model’s performance by modifying of its receptive field excitatory and inhibitory subfields, we formulated possible receptive field abnormalities in patients. The formulated model receptive field changes (blue) were compared to the base (control) model receptive field profiles (black). Formulated changes for medicated patients include an increase in the spread (width) and strength (height) of the inhibitory subfield (**E**), increase in the spread (width) of the excitatory and inhibitory subfields (**F**), or decrease in the strength (amplitude) of the excitatory and inhibitory subfields (**G**). Formulated changes for unmedicated patients include decrease in the spread (width) and strength (amplitude) of the inhibitory subfield (**H**) or decrease in the spread (width) and increase in the strength (amplitude) of the excitatory subfield (**I**). Based on currently available data, each formulated change was cross-referenced with previous studies’ stimulus and experiment type to determine whether it would be more related to the magnocellular or parvocellular system. **E** Prior studies finding decreased sensitivity at low spatial frequencies in medicated patients suggested primarily magnocellular involvement^3,5–8^ although a small number suggested parvocellular involvement^5,9^. **F** Prior studies finding decreased sensitivity at medium-high spatial frequencies in medicated patients suggested primarily parvocellular involvement^4,10^, although magnocellular involvement may be possible^4^. **G** Prior studies finding decreased sensitivity across spatial frequencies in medicated patients suggest both magnocellular^4,10^ and parvocellular^4,9^ involvement. **H** Prior studies finding increased sensitivity at low spatial frequencies in unmedicated patients suggested magnocellular involvement^5,6,11,12^. **I** *Although available data involving unmedicated patients is limited and mostly related to low spatial frequencies, the general trend of increased sensitivity in unmedicated patients is hypothesized to be potentially related to involvement of both the magnocellular and parvocellular system. Prior studies suggest magnocellular involvement in unmedicated patients in the low spatial frequency range^5,6,11,12^, although future studies would be needed to investigate results and system involvement for medium and high spatial frequencies in unmedicated patients. The possibility of generalized increased sensitivity in unmedicated patients can be fitted/replicated in Fig. 2I, J.

When the excitatory and inhibitory subfield extents were both decreased, the model most notably demonstrated increased sensitivity at medium spatial frequencies. When the excitatory and inhibitory subfield extents were both increased, the model demonstrated decreased sensitivity across spatial frequencies. To determine the impact of the receptive field and its excitatory/inhibitory subfield extents, the raw values of *σ*_*ex*_ and *σ*_*inh*_ were altered, but the base ratio of 1:1.6 was maintained (Fig. 4A–D). *Amp*_*inh*_ and *Amp*_*ex*_ were both held at values of 1. To determine the effect of a reduced receptive field size, σ_ex_ and σ_inh_ were both altered by a factor of 0.9 (Fig. 4A, B, blue). Under this condition, the model showed most pronounced increased sensitivity in the medium spatial frequency range, than in low and high spatial frequencies. To determine the effect of an increased receptive field size, *σ*_*ex*_ and *σ*_*inh*_ were both altered by a factor of 1.1 (Fig. 4A, B, red). Under this condition, the model demonstrated overall decreased sensitivity. In both conditions in which the ratio of *σ*_*ex*_ to *σ*_*inh*_ was maintained at 1:1.6, the contrast sensitivity curves and receptive field profiles demonstrated similar shapes.

#### Maintaining the ratio of excitatory (*Amp*_*ex*_) and inhibitory (*Amp*_*inh*_) subfields amplitudes

When the excitatory and inhibitory subfield amplitudes were both decreased, the model exhibited decreased sensitivity across spatial frequencies. When the excitatory and inhibitory subfield amplitudes were both increased, the model demonstrated increased sensitivity across spatial frequencies. To determine the effects of the ratio of excitatory to inhibitory subfields amplitude, *σ*_*ex*_ and *σ*_*inh*_ were maintained at base values of 1 and 1.6, respectively. The raw values of *Amp*_*ex*_ and *Amp*_*inh*_ were altered, while keeping the 1:1 ratio of *Amp*_*ex*_/ *Amp*_*inh*_ (Fig. 4C, D). When the values of *Amp*_*ex*_ and *Amp*_*inh*_ were decreased to 0.5, maintaining the same ratio, the model demonstrated overall decreased sensitivity across spatial frequencies. However, the model did not show a significant shift in the spatial frequency at which maximum sensitivity occurred (Fig. 4C, D, blue). When the values of *Amp*_*ex*_ and *Amp*_*inh*_ were increased to 1.5, maintaining the same 1:1 ratio, the model demonstrated overall increased sensitivity across spatial frequencies. The spatial frequency at which maximum sensitivity occurred was also shifted to a lower value (Fig. 4C, D, red). When the ratio of *Amp*_*ex*_/ *Amp*_*inh*_ was maintained, the shape of the resulting spatial frequency sensitivity curves resembled that of the base control curve.

## Discussion

In this work, we used a neural model of on-center/off-surround neurons in the early visual system to replicate spatial frequency sensitivity abnormalities reported in perceptual data from medicated and unmedicated patients with schizophrenia (Tables 2–3 and supplementary Table 1). In order to have the model replicate contrast sensitivity changes in medicated and unmedicated schizophrenia patients we tried all combinations of parameter changes related to excitatory and inhibitory subfields and selected those that replicated empirical data of contrast sensitivity abnormalities (Tables 2–3, supplementary Table 1, summarized in Fig. 4E–I). Based on the model results, we hypothesize that medicated patients may have an increase in the width (spread) and height (strength) of the inhibitory subfield, or either an increase in width (spread) or decrease in amplitude (strength) of the excitatory and inhibitory subfields (Fig. 4E–G). Unmedicated patients may display a decrease in the width and amplitude of the inhibitory subfield or a decrease in the width and increase in the amplitude of the excitatory subfield (Fig. 4H, I).

**Table 3:** Studies of spatial frequency sensitivity using static and dynamic visual stimuli differentially involving parvocellular and magnocellular pathways in medicated and unmedicated patients with schizophrenia.

Stimulus type	Stimuli tested	Reference	Medication status	Spatial frequency range affected	Pathway relative involvement	Corresponding model results
Dynamic (Moving)	Vertical luminance contrast sinusoidal grating	Chen et al. 2003^6^	atypical, typical, and no antipsychotics	Low	Magnocellular	Medicated: ↑ *σ*_*inh*_ & ↑ *Amp*_*inh*_ Unmedicated: ↓ *σ*_*inh*_ & ↓ *Amp*_*inh*_
Dynamic (Moving, Phase Shift) 2AFC	Horizontal luminance sinusoidal grating stimuli	Cadenhead et al. 2013^5^	Medicated and unmedicated patients	Low	Magnocellular	Medicated: ↑ *σ*_*inh*_ & ↑ *Amp*_*inh*_ Unmedicated: ↓ *σ*_*inh*_ & ↓ *Amp*_*inh*_
Dynamic (Moving, Phase Shift) 2AFC	Horizontal chromatic (red/green) sinusoidal grating stimuli	Cadenhead et al., 2013^5^	Medicated and unmedicated patients	Low (Both medicated and unmedicated patients showed decreased sensitivity to chromatic contrast)	Parvocellular	↑ *σ*_*inh*_ & ↑ *Amp*_*inh*_
Dynamic (Phase reversal) 2AFC	Horizontal sinusoidal contrast grating stimuli	Cimmer et al 2006^8^	Medicated	Low	Magnocellular	↑ *σ*_*inh*_ & ↑ *Amp*_*inh*_
Dynamic (Phase Reversal)	Horizontal sinusoidal grating stimuli	Kéri et al. 2002^10^	Medicated	Low-high	Magnocellular	↓ *Amp*_*ex*_ & ↓ *Amp*_*inh*_
Dynamic (Drifting)	Vertical sinusoidal gratings	Slaghuis 1998^4^	Medicated	Low-high*	Magnocellular	↓ Amp_ex_ & ↓ Amp_inh_*
Static	Horizontal sinusoidal grating stimuli	Kéri et al. 2002^10^	Medicated	Medium-high	Parvocellular	↑ *σ*_*ex*_ & ↑ *σ*_*inh*_
Static	Vertical sinusoidal gratings	Slaghuis 1998^4^	Medicated*	Low-high*	Parvocellular	↓ *Amp*_*ex*_ & ↓ *Amp*_inh_*
Static (2AFC)	Luminance contrast stimuli	Butler et al. 2005^3^	Medicated	Low-medium	Magnocellular	↑ *σ*_*inh*_ & ↑ *Amp*_*inh*_
Static (2AFC)	Horizontal sinusoidal gratings	Butler et al. 2009^7^	Medicated	Low	Magnocellular	↑ *σ*_*inh*_ & ↑ *Amp*_*inh*_
Static (Orientation detection)	“Pulsed pedestal” Sinusoidal grating Gabor patches	Kiss et al. 2010^11^	Unmedicated	No significant difference	Parvocellular	No difference noted
Static (Orientation detection)	“Steady pedestal” Sinusoidal grating Gabor patches	Kiss et al. 2010^11^	Unmedicated	Low	Magnocellular	↓ *σ*_*inh*_ & ↓ *Amp*_*inh*_
Static (Orientation detection)	“Pulsed pedestal” Sinusoidal grating Gabor patches	Kelemen et al. 2013^12^	Participants unmedicated at baseline	No significant difference	Parvocellular	No difference noted
Static (Orientation detection)	“Steady pedestal” Sinusoidal grating Gabor patches	Kelemen et al. 2013^12^	Participants unmedicated at baseline	Low	Magnocellular	↓ *σ*_*inh*_ & ↓ *Amp*_*inh*_
Static (Threshold detection)	Gabor elements	Shoshina and Shelepin, 2015^9^	Medicated	SZ patients < 10 years: low SZ patients > 10 years: Low-high	Parvocellular	SZ patients < 10 years: ↑ *σ*_*inh*_ & ↑ *Amp*_*inh*_ SZ patients > 10 years: ↓ *Amp*_*ex*_ & ↓ *Amp*_*inh*_

By comparing the medication status of subjects in each study along with the spatial frequency range at which effects were observed, each study was matched with the corresponding model alterations from Fig. 4.

*SZ* Schizophrenia, *2AFC* Two-Alternative Forced Choice

*For negative symptom SZ. For positive symptom SZ, reductions were only seen at medium and high spatial frequencies, and the corresponding model results would instead be ↑ *σ*_ex_ and ↑ *σ*_inh._.

Given the nature of the present model, it is possible to consider excitatory and inhibitory subfield effects separately and evaluate how those effects change the overall receptive field properties. For instance, changes to an inhibitory neurotransmitter may alter only the spread and amplitude of inhibition but not excitation, and would still change the overall nature of the receptive field organization. For a case in which both the spread and amplitude of a subfield change, it could stem from synaptic pruning^56^, changes in density of receptors^57–61^, a decrease or increase in the number of neurons or interneurons^62,63^. Changes in amplitude could result from hypo- or hyperactivity in certain neurons due to similar factors^17,26,64^. For cases in which there are opposite changes in amplitude and spread, like the case in which the excitatory subfield width decreases while the amplitude increases, this could possibly indicate compensatory mechanisms^65–70^. For instance, excitatory neuron pruning could lead to a decrease in subfield width, but compensatory mechanisms could increase neuronal activity and thus increase amplitude. Whether these changes occur in inhibitory or excitatory neurons would differentially affect either the excitatory or inhibitory subfields, thus having a variable impact on the overall receptive field organization.

Additionally, there is much left to be understood about the pathophysiology of schizophrenia and the abnormalities which may contribute to receptive field organization and connectivity changes. For instance, further studies investigating myelination and conduction velocity in schizophrenia would provide valuable insight into the pathophysiology of the disorder. It is possible that conduction velocity and myelination, besides the axonal sprouting and dendritic arborization abnormalities, could alter the effective amplitude of excitation and inhibition as well as their extent. Additionally, although interneuron abnormalities have been suggested to play a role in the pathophysiology of schizophrenia (reviewed in ref. ^71^), further research is needed to discover the specific subtypes of interneurons that may be involved in different brain areas and networks. Involvement of different types of interneurons could have varying effects on either the spread or amplitude of inhibition, thus contributing to receptive field abnormalities observed in these patients.

Certain elements of the pathophysiology of schizophrenia could manifest differently over the course of illness. For instance, antipsychotic medication is not likely to result in connectivity pruning in the short-term, such as in the case of patients who were on medication for a short period of time and demonstrated spatial frequency sensitivity similar to that of controls^12^. However, before long-term synaptic connectivity changes occur, it is possible that medication administration would change certain neurochemical interactions, thus leading to modification in synaptic gain and an effective change in subfield amplitude. At later stages, with increased pruning, it is possible that the subfield width could also be affected, leading to coupled effects of width and amplitude over time. Therefore, initial alteration of one aspect of the receptive field subfield can lead to generalized, coupled alterations of all receptive field characteristics.

Below we discuss these findings in the context of the major theories regarding the pathophysiology of schizophrenia and the mechanism of action of common antipsychotic medications. In particular, we correlate our findings with dopaminergic, glutamatergic, and GABAergic abnormalities that have been implicated in the disorder^13–27^, the presentation of positive, negative, or cognitive symptoms^20^, and the type of antipsychotic medications, which can be classified as either typical, first-generation dopaminergic receptor antagonists, or atypical, second-generation targeting several receptors^72,73^. The current findings consolidate a broad range of experiments using a variety of stimuli that can differentially involve parvocellular and magnocellular pathways. For each scenario, we used the amplitude and the extent of excitation and inhibition fitting to compute possible outputs within each subsystem, shown in Fig. 4E–I, summarizing the overall impacts of medicated and unmedicated states in magnocellular and parvocellular systems, respectively. While many studies suggest an impact on the magnocellular system^3,4,7,11,12^, it is also possible that the parvocellular system is affected^4,5,10^ given perceptual data in response to more parvocellular-biased stimuli. However, it is possible that the parvocellular system remains intact for unmedicated patients^11,12^, but not in medicated patients at later stages of the disease^9^. Overall, the RF change reverse engineered by this model can be summarized based on Fig. 4E–I as follows: in *unmedicated status, the* extent and amplitude of excitation goes up for the magnocellular system whereas, the parvocellular system may escape from such changes^11,12^, and in *medicated status*, both magnocellular and parvocellular systems, depending on their relative involvement, based on the stimulus type used, show imbalance of excitation/inhibition ratios with specific reductions in amplitude and extent. These functional findings in the context of balance of inhibition and excitation in the two subsystems offer novel insights for targeted studies that can examine specific connectivity or neurotransmitter changes in schizophrenia, including density of neurotransmitter channels, axonal sprouting and myelination, dendritic arborization, density, distribution, and ratio of distinct interneuron types, and synaptic interactions. These can be examined both in visual brain networks and in other cortical and subcortical regions, beyond the visual system, including different thalamic nuclei to examine sensory and association processing in schizophrenia, through biochemical, pharmacological, electrophysiological, neuroimaging, and postmortem neuroanatomical approaches.

Studies showed that medicated patients with schizophrenia exhibit variable decreases in sensitivity across a wide range of spatial frequencies, showing deficits either at low^3,5–9^, or medium/high^4,10^, or across all frequencies^4,9,10^. At low spatial frequencies, increased levels of inhibition best replicated medicated patient perceptual data, in line with evidence supporting increased inhibition in the cortex^74^ and impaired inhibitory signaling^75^. Moreover, our model highlighted one plausible mechanism to achieve decreased sensitivity at medium/high spatial frequencies that is in line with the observed reduction of inhibitory surround in population receptive field in medicated patients with schizophrenia^76^: simultaneous alterations of the excitatory and inhibitory spread with a more dramatic increase in inhibition, so that the ratio of the subfields was not constant. In contrast, multiple parameter combinations resulted in a reduction in the model’s sensitivity across spatial frequencies, in line with behavioral findings and hypotheses based on experimental data^5^. Decreasing the amplitude of excitation and inhibition, which is consistent with studies that have reported reduced interneuron activity and excessive excitatory pruning in schizophrenia^77,78^, provided the best fit. Dampening of the excitatory amplitude, is also supported by new findings that reductions in excitatory synaptic gain, may be linked to the pathophysiology of schizophrenia^79^. Lastly, narrower model inhibition or wider excitation caused imbalance and lower model sensitivity that may reflect altered center-surround interactions in patients with schizophrenia^80^.

Unmedicated patients with schizophrenia, on the other hand, exhibit the opposite symptoms, demonstrating increases in spatial frequency sensitivity either at low^5,6,11,12^, or what is considered to be generally increased spatial frequency sensitivity. An overall decrease in inhibition, best replicated these findings for low spatial frequencies in the simulations, and an increase in model excitation replicated the general trend in perceptual data across frequencies. A reduction in the spread and strength of inhibition would be consistent with reduced inhibitory GABAergic interneuron function due to NMDA receptor dysfunction that could lead to increased excitatory glutamatergic signaling downstream^21,27^. The model best replicated the general trend of increased sensitivity in unmedicated patients with schizophrenia when the strength of excitation increased but the spread decreased. Increased cortical excitability in unmedicated patients could reflect an increase in the receptive field’s excitatory amplitude^81^, while a decrease in excitatory breadth could reflect pruning of excitatory synapses, hypothesized in schizophrenia^77^. The model was able to replicate the range of spatial frequency changes in unmedicated patients (Fig. 4H, I).

Results from previous studies have indicated the possibility that medication type and dosage may alter spatial frequency sensitivity in patients with schizophrenia, affecting perception^3^. For instance, in medicated patients that exhibited decreased contrast sensitivity at medium and high spatial frequencies, increased antipsychotic dosage was associated with more severe deficits in contrast sensitivity^10^. Medicated and unmedicated patients that were tested at a low spatial frequency (0.5 cpd) did not exhibit significant changes compared to controls, when taking atypical antipsychotics, but demonstrated decreased sensitivity when taking typical medication, or increased sensitivity, if not medicated^6^. Other studies have corroborated the finding that decreased sensitivity is seen in patients taking first-generation antipsychotics^9^, but some studies found decreased spatial frequency sensitivity even in patient groups taking mostly second-generation antipsychotics^8^. The model was able to replicate the entire range of potential changes in medicated patients (Fig. 4E–I).

Because, first-generation antipsychotics are dopamine D2 receptor antagonists, and their effectiveness is correlated with their D2 receptor binding capacity^72^, it is possible that the spatial frequency deficits seen in medicated patients are a result of deficient dopamine signaling. Typical antipsychotics can cause a condition known as drug-induced Parkinsonism, in which dopamine antagonism produces symptoms like those seen in Parkinson’s disease^82^. Patients with Parkinson’s tend to exhibit decreased contrast sensitivity at a range of spatial frequencies, but these effects can be mitigated by drugs such as levodopa, possibly implicating dopamine in contrast sensitivity deficits^83,84^. Dopamine may also have an effect as early as the retina, where it can weaken gap junctions between horizontal cells, thus reducing receptive field size^25,85^. In medicated patients, decreased dopamine could lead to an increase in receptive field size and thus decreased spatial frequency sensitivity. If, in unmedicated patients, dopamine signaling is increased at the level of the retina, this could result in a decreased receptive field size and, thus, increased spatial frequency sensitivity.

Thus, if medication type is considered in the context of spatial frequency sensitivity, it is possible that dopamine antagonism produces contrast sensitivity deficits, whereas second-generation antipsychotic medications which have a comparatively lower affinity for dopamine receptors do not produce these same deficits. In addition, antipsychotic medications decrease glutamate metabolite levels over the course of treatment, thus providing a potential insight into the differences observed in spatial frequency sensitivity between medicated and unmedicated patients^23^. Therefore, when comparing perceptual results in medicated patients with those from unmedicated patients, it is important to take note of the medication regimens, as this could present a key variable.

Illness duration could play a key role in the spatial frequency heterogeneity observed in medicated and unmedicated patients with schizophrenia, given that patients tend to be unmedicated earlier in the course of the illness. Previous studies have indeed observed that illness duration affects spatial frequency sensitivity in medicated patients with schizophrenia^9^. However, studies have also shown that administration of antipsychotic medication mitigates the increased spatial frequency sensitivity seen in unmedicated patients, bringing it to levels observed in control participants^12^. Thus, antipsychotic medication appears to play a role in spatial frequency sensitivity patterns seen in patients with schizophrenia, despite the fact that illness duration may affect spatial frequency sensitivity as well. Perhaps the increased spatial frequency sensitivity observed in unmedicated patients is related to the initial pathophysiology of the disorder itself, whereas the decreased spatial frequency sensitivity seen in medicated patients is the result of an interplay between antipsychotic medication effects as well as illness course and progression. In line with this, studies in healthy individuals or animals have shown that selective D2 receptor antagonists may change the amplitudes of event-related potentials in the auditory^86^ and visual systems^87^. D2R antagonism has been shown to affect spatial working memory and planning in humans^88,89^, executive function in primates^90^, visuo-cognitive^87^, and cognitive processes^86^. Future studies investigating the role of dopamine D2 receptor (D2R) blockade in healthy controls would help further elucidate the precise role of antipsychotic medication in spatial frequency sensitivity. Additional animal studies investigating the effect of antipsychotic medication and D2R blockade on the visual system and other sensory modalities would be a valuable area of research.

The stimulus types utilized to test observers, whether static, with and without phase reversal, dynamic, with drift, motion, chromatic, achromatic, etc., could influence the range of observed spatial frequency sensitivity abnormalities (Table 3). This suggests that the studies reviewed in Table 1 can be approached within the context of key stimuli characteristics, that can differentially influence visual parvocellular and magnocellular subsystems. For instance, some studies utilized stimuli with a motion component^4–6^, whereas other studies used stationary stimuli^3,7–12^. Of the studies that used stationary stimuli, some did not incorporate a temporal element^3,7,11,12^, whereas other studies incorporated phase reversal^8^. One study compared responses between spatially static stimuli with and without phase reversal^10^, and another study compared responses when stimuli were stationary and drifting^4^. Using a contrast grating stimulus with and without phase reversal, could reveal spatial frequency sensitivity deficit across spatial frequencies^10^ with the added context that the contrast sensitivity has been tested by static and dynamic (with phase reversal) stimuli, biasing the involvement of different visual subsystems. Although the range at which abnormalities were observed varied, the overall direction of the contrast sensitivity changes remained the same. When comparing studies that have used the similar type of stimuli, similar patterns emerge, i.e., medicated patients exhibit decreased contrast sensitivity and unmedicated patients exhibit increased contrast sensitivity.

The range of spatial frequencies affected could depend on the relative involvement of the magnocellular and parvocellular system, based on the stimulus type. Previous studies have shown that patients with schizophrenia demonstrate abnormalities in magnocellular pathway processing^91–93^, and other studies used stimuli with biased involvement of magnocellular and parvocellular systems^3–5,7,10–12^. Overall, these studies suggest that the contrast sensitivity deficit in schizophrenia likely involves disruptions mostly, but not exclusively, in the magnocellular system whereas, disruptions in the parvocellular system, even though not as pervasive, are noted more in medicated patients^4,5,10^ than in unmedicated patients^11,12^.

The spatial, contrast level, and temporal aspects of stimuli in some of these studies were modulated in such a way to selectively bias targeting of either magnocellular or parvocellular processing and found that deficits in spatial frequency sensitivity for medicated patients were more prevalent with magnocellular-biased stimuli^3,7^. Also a similar pattern emerges for unmedicated patients, who demonstrated increased sensitivity at low spatial frequencies in magnocellular-biased trials, but not in parvocellular-biased trials^11,12^. Studies which utilized stimuli with a motion or drifting component, which would most likely affect or bias processing by the magnocellular system, found effects in the low spatial frequency range^4,6^, and also in other frequency ranges (mid and high) both in magnocellular- and parvocellular-biased stimuli (Table 3). Thus, it is possible that the magnocellular system is most directly affected, contributing to the low-level visual processing abnormalities seen in patients with schizophrenia, yet the parvocellular system may not be completely spared. Given that the magnocellular system is responsible for conveying low spatial frequency information^94,95^, this could potentially explain the pattern of abnormalities seen in medicated and unmedicated patients at low spatial frequencies. While some researchers have hypothesized that the magnocellular pathway in patients with schizophrenia is hypoactive^96,97^, others have hypothesized that this pathway may be overactive in unmedicated patients^12^. From a modeling perspective, the respective hypo- or hyperactivity of the magnocellular system could manifest as an effective change in the average population receptive field qualities, thus leading to alterations in spatial frequency sensitivity. Further studies would be needed to confirm the spatial frequency effects on unmedicated and long-term medicated patients for magnocellular vs. parvocellular-biased stimuli, and our computational modeling platform, can provide a useful framework to design and interpret new findings.

Importantly, NMDA receptors reach their maximum density in the primary visual cortex, where dopaminergic D1, GABA_A_, and GABA_A_/BZ receptors are also particularly dense^98^. It is therefore likely that glutamatergic NMDA receptor hypofunction, GABAergic dysfunction, and dopamine dysregulation in V1 interact to contribute to visual processing abnormalities^17,26^ affecting both unmedicated and medicated patients^99,100^. Prior studies using animal models have found that ketamine, an NMDA receptor antagonist, induces electrophysiological changes in V1^101^. Other studies have found that ketamine alters neuron orientation selectivity in V1, suggesting that NMDA receptor abnormalities would have an impact on neural response properties in V1^102^. These changes, which are hypothesized to be linked to V1 neural plasticity, affect both individual neurons and overall connectivity between neurons^88^, which lends further credit to the hypothesis that there may be population receptive field changes in patients with schizophrenia given the implication of glutamate and NMDA receptors in the pathophysiology of the disorder. Additionally, NMDA receptors have been shown to have a substantial impact on contrast responses in certain lower visual system cell types, which would indicate that disruption to this signaling would affect these responses^103^. Moreover, related to further neurotransmitter abnormalities, GABA-related gene expression is altered in patients with schizophrenia^15,16^ and GABA concentration is reduced in V1 of patients with schizophrenia, and this reduction is associated with dysfunction in orientation-specific surround suppression^17,18^.

### Limitations and simplifications

Our parameterized, rate-based neural model represents a simplification of various early visual areas, such as the primary visual cortex (V1), association visual cortices V2, V4, and the lateral geniculate nucleus (LGN). The single layer model could represent an ensemble of multiple visual areas and their accumulated activities. Since the model is single layer, it is also important to note that it can only approximate network changes that may be occurring in schizophrenia, due to the thalamocortical loop connectivities or those relating to feedback mechanisms^104^. Given that contrast sensitivity is a relatively low-level visual process, this simplification could be a good starting point model for an initial investigation of the underlying mechanisms of related abnormalities seen in schizophrenia^32^. This is relevant given that spatial frequency processing involves both feedforward and recurrent/feedback processing^105–107^. Although the model does not directly incorporate feedback mechanisms, these elements could be partially underlying broader changes in excitation and inhibition that we examined. With the understanding that the model still represents a simplified version of complicated sensory mechanisms, the model’s “receptive field” is actually a type of population receptive field. This allows changes across different visual areas to be incorporated into a single model layer representing the interconnected areas. This method simplifies the population receptive field properties into *σ* (width, spread) and amplitude (height, strength), thus allowing for the investigation of abnormalities that arise from a variety of detailed neurobiological mechanisms, which impact the inhibitory and excitatory subfields. Therefore, neurotransmitter and receptor-related abnormalities could be simplified, incorporated, and interpreted in terms of changes in excitation and inhibition width/amplitude. Because contrast sensitivity is heavily dependent on the relative balance of the excitatory and inhibitory subfields, this simplified model can provide targeted and valuable insights into contrast sensitivity abnormalities in patients with schizophrenia that arise from changes in one or simultaneous manipulations of several parameters.

## Conclusions

The rate-based, feedforward model we developed demonstrates that the spatial frequency sensitivity abnormalities observed in medicated and unmedicated patients with schizophrenia can be replicated in terms of alterations to the excitatory and inhibitory receptive field subfields. The results indicate that medicated patients with schizophrenia may have increased neural inhibition, altered receptive field size, or concurrently altered levels of excitation and inhibition. Unmedicated patients, on the other hand, may have either increased excitation or decreased inhibition. By utilizing this technique to model and explore the possible pathophysiology of schizophrenia, it is possible to make connections between the hypotheses that exist about this disease and other factors that may influence the observed perceptual deficits.

### Supplementary information

Supplementary tables

Simulation code

## Data Availability

The original contributions presented in the study are included in the article/supplementary material, further inquiries can be directed to the corresponding authors.

## References

[1] Javitt DC (2009). Sensory processing in Schizophrenia: Neither simple nor intact. Schizophr. Bull..

[2] Diamond A, Silverstein SM, Keane BP (2022). Visual system assessment for predicting a transition to psychosis. Transl. Psychiatry.

[3] Butler PD (2005). Early-stage visual processing and cortical amplification deficits in Schizophrenia. Arch. Gen. Psychiatry.

[4] Slaghuis WA (1998). Contrast sensitivity for stationary and drifting spatial frequency gratings in positive- and negative-symptom schizophrenia. J. Abnorm Psychol..

[5] Cadenhead K. S., Dobkins K., McGovern J., Shafer K. Schizophrenia spectrum participants have reduced visual contrast sensitivity to chromatic (red/green) and luminance (light/dark) stimuli: new insights into information processing, visual channel function, and antipsychotic effects. *Front Psychol*. 4 10.3389/fpsyg.2013.00535 (2013). 10.3389/fpsyg.2013.00535 PMC3747446 23970874

[6] Chen Y (2003). Effects of typical, atypical, and no antipsychotic drugs on visual contrast detection in Schizophrenia. Am. J. Psychiatry.

[7] Butler PD (2009). Sensory contributions to impaired emotion processing in Schizophrenia. Schizophr. Bull..

[8] Cimmer C (2006). Abnormal neurological signs, visual contrast sensitivity, and the deficit syndrome of schizophrenia. Prog. Neuro-Psychopharmacol. Biol. Psychiatry.

[9] Shoshina II, Shelepin YE (2015). Contrast sensitivity in patients with Schizophrenia of different durations of illness. Neurosci. Behav. Physiol..

[10] Kéri S, Antal A, Szekeres G, Benedek G, Janka Z (2002). Spatiotemporal visual processing in Schizophrenia. J. Neuropsychiatry Clin. Neurosci..

[11] Kiss I, Fábián Á, Benedek G, Kéri S (2010). When doors of perception open: Visual contrast sensitivity in never-medicated, first-episode schizophrenia. J. Abnorm Psychol..

[12] Kelemen O, Kiss I, Benedek G, Kéri S (2013). Perceptual and cognitive effects of antipsychotics in first-episode schizophrenia: The potential impact of GABA concentration in the visual cortex. Prog. Neuro-Psychopharmacol. Biol. Psychiatry.

[13] Liu Y. et al. A selective review of the excitatory-inhibitory imbalance in schizophrenia: underlying biology, genetics, microcircuits, and symptoms. *Front Cell Dev. Biol*. 9 10.3389/fcell.2021.664535 (2021). 10.3389/fcell.2021.664535 PMC8567014 34746116

[14] Howes O, McCutcheon R, Stone J (2015). Glutamate and dopamine in schizophrenia: An update for the 21st century. J. Psychopharmacol..

[15] Hashimoto T (2008). Conserved regional patterns of GABA-related transcript expression in the neocortex of subjects with schizophrenia. Am. J. Psychiatry.

[16] Egerton A, Modinos G, Ferrera D, McGuire P (2017). Neuroimaging studies of GABA in schizophrenia: A systematic review with meta-analysis. Transl. Psychiatry.

[17] Yoon JH (2020). Reduced in vivo visual cortex GABA in schizophrenia, a replication in a recent onset sample. Schizophr. Res.

[18] Yoon JH (2010). GABA concentration is reduced in visual cortex in schizophrenia and correlates with orientation-specific surround suppression. J. Neurosci..

[19] Gonzalez-Burgos G, Lewis DA (2008). GABA neurons and the mechanisms of network oscillations: implications for understanding cortical dysfunction in schizophrenia. Schizophr. Bull..

[20] Patel, K. R., Cherian, J., Gohil, K. & Atkinson, D. Schizophrenia: overview and treatment options. *P T* **39**, 638–645, http://www.ncbi.nlm.nih.gov/pubmed/25210417 (2014). PMC4159061 25210417

[21] Snyder MA, Gao W-J (2013). NMDA hypofunction as a convergence point for progression and symptoms of Schizophrenia. Front Cell Neurosci..

[22] Kayser MS, Dalmau J (2016). Anti-NMDA receptor encephalitis, autoimmunity, and psychosis. Schizophr. Res.

[23] Merritt K (2019). Remission from antipsychotic treatment in first episode psychosis related to longitudinal changes in brain glutamate. NPJ Schizophr..

[24] Brisch R. et al. Corrigendum: The role of dopamine in Schizophrenia from a neurobiological and evolutionary perspective: Old fashioned, but still in vogue. *Front Psychiatry*. 5 10.3389/fpsyt.2014.00110 (2014). 10.3389/fpsyt.2014.00047 PMC4032934 24904434

[25] Silverstein SM, Rosen R (2015). Schizophrenia and the eye. Schizophr. Res. Cogn..

[26] McCutcheon RA, Krystal JH, Howes OD (2020). Dopamine and glutamate in schizophrenia: Biology, symptoms and treatment. World Psychiatry.

[27] Olney JW, Newcomer JW, Farber NB (1999). NMDA receptor hypofunction model of Schizophrenia. J. Psychiatr. Res.

[28] Rassovsky Y, Horan WP, Lee J, Sergi MJ, Green MF (2011). Pathways between early visual processing and functional outcome in schizophrenia. Psychol. Med.

[29] Schiffman J (2006). Premorbid childhood ocular alignment abnormalities and adult schizophrenia-spectrum disorder. Schizophr. Res.

[30] Schubert EW, Henriksson KM, McNeil TF (2005). A prospective study of offspring of women with psychosis: Visual dysfunction in early childhood predicts schizophrenia‐spectrum disorders in adulthood. Acta Psychiatr. Scand..

[31] Silverstein S (2015). Vision in schizophrenia: Why it matters. Front Psychol..

[32] Butler PD (2007). Subcortical visual dysfunction in Schizophrenia drives secondary cortical impairments. Brain.

[33] Yoon JH, Sheremata SL, Rokem A, Silver MA (2013). Windows to the soul: vision science as a tool for studying biological mechanisms of information processing deficits in Schizophrenia. Front Psychol..

[34] Callaway EM (1998). Local circuits in primary visual cortex of the Macaque Monkey. Annu Rev. Neurosci..

[35] Angelucci A (2002). Circuits for local and global signal integration in primary visual cortex. J. Neurosci..

[36] Hirabayashi T, Miyashita Y (2014). Computational principles of microcircuits for visual object processing in the macaque temporal cortex. Trends Neurosci..

[37] Gilbert CD (1983). Microcircuitry of the visual cortex. Annu Rev. Neurosci..

[38] Irvin GE, Casagrande VA, Norton TT (1993). Center/surround relationships of magnocellular, parvocellular, and koniocellular relay cells in primate lateral geniculate nucleus. Vis. Neurosci..

[39] Cavanaugh JR, Bair W, Movshon JA (2002). Nature and interaction of signals from the receptive field center and surround in Macaque V1 neurons. J. Neurophysiol..

[40] Angelucci A., Bressloff P. C. Contribution of feedforward, lateral and feedback connections to the classical receptive field center and extra-classical receptive field surround of primate V1 neurons. *Vis Percept - Fundam Vis Low Mid-Level Process Percept*. 93–120 10.1016/s0079-6123(06)54005-1 (2006). 10.1016/S0079-6123(06)54005-1 17010705

[41] Angelucci A., Levitt J. B., Lund J. S. Chapter 29 Anatomical origins of the classical receptive field and modulatory surround field of single neurons in macaque visual cortical area V1. *Prog Brain Res*. 373–388 10.1016/s0079-6123(02)36031-x (2002). 10.1016/s0079-6123(02)36031-x 12143395

[42] Hilgetag CC, Medalla M, Beul SF, Barbas H (2016). The primate connectome in context: Principles of connections of the cortical visual system. Neuroimage.

[43] Layton O. W., Mingolla E., Yazdanbakhsh A. Dynamic coding of border-ownership in visual cortex. *J. Vis*. 10.1167/12.13.8 (2012). 10.1167/12.13.8 23220579

[44] Layton O. W., Yazdanbakhsh A. A neural model of border-ownership from kinetic occlusion. *Vision Res*. 10.1016/j.visres.2014.11.002 (2015). 10.1016/j.visres.2014.11.002 25448117

[45] Layton O. W., Mingolla E., Yazdanbakhsh A. Neural dynamics of feedforward and feedback processing in figure-ground segregation. *Front Psychol*. 10.3389/fpsyg.2014.00972 (2014). 10.3389/fpsyg.2014.00972 PMC4193330 25346703

[46] Sherbakov L., Yazdanbakhsh A. Multiscale sampling model for motion integration. *J Vis*. 10.1167/13.11.18 (2013). 10.1167/13.11.18 24080519

[47] Park S, Zikopoulos B, Yazdanbakhsh A (2022). Visual illusion susceptibility in autism: A neural model. Eur. J. Neurosci..

[48] Zhu J., Zikopoulos B., Yazdanbakhsh A. A neural model of modified excitation/inhibition and feedback levels in Schizophrenia. *Front Psychiatry*. 14 https://www.frontiersin.org/articles/10.3389/fpsyt.2023.1199690 (2023). 10.3389/fpsyt.2023.1199690 PMC10600455 37900297

[49] Hubel DH, Wiesel TN (1968). Receptive fields and functional architecture of monkey striate cortex. J. Physiol..

[50] Gilbert CD, Wiesel TN (1992). Receptive field dynamics in adult primary visual cortex. Nature.

[51] Olson SJ, Grossberg S (1998). A neural network model for the development of simple and complex cell receptive fields within cortical maps of orientation and ocular dominance. Neural Netw..

[52] Bruno RM, Simons DJ (2002). Feedforward mechanisms of excitatory and inhibitory cortical receptive fields. J. Neurosci..

[53] Grossberg S (1973). Contour enhancement, short term memory, and constancies in reverberating neural networks. Stud. Appl Math..

[54] Yazdanbakhsh A, Layton O, Mingolla E (2012). A neural model of border-ownership and motion in early vision. J. Vis..

[55] Marr D, Hildreth E (1980). Theory of edge detection. Proc. R. Soc. Lond. - Biol. Sci..

[56] Sellgren CM (2019). Increased synapse elimination by microglia in schizophrenia patient-derived models of synaptic pruning. Nat. Neurosci..

[57] Kim S. A. 5-HT1A and 5-HT2A Signaling, desensitization, and downregulation: Serotonergic dysfunction and abnormal receptor density in Schizophrenia and the prodrome. *Cureus*. 10.7759/cureus.15811 (2021). 10.7759/cureus.15811 PMC8294605 34306878

[58] Buchsbaum M (2006). D2/D3 dopamine receptor binding with [F-18] fallypride in thalamus and cortex of patients with Schizophrenia. Schizophr Res..

[59] Lehrer DS (2010). 18F-Fallypride binding potential in patients with Schizophrenia compared to healthy controls. Schizophr. Res..

[60] Kessler RM (2009). Dopamine D2 receptor levels in striatum, thalamus, substantia nigra, limbic regions, and cortex in Schizophrenic subjects. Biol. Psychiatry.

[61] Kegeles LS (2010). Striatal and extrastriatal dopamine D2/D3 receptors in Schizophrenia Evaluated with [18F]fallypride positron emission tomography. Biol. Psychiatry.

[62] Gaus R (2023). Reduced cortical neuron number and neuron density in schizophrenia with focus on area 24: a post-mortem case–control study. Eur. Arch. Psychiatry Clin. Neurosci..

[63] Kreczmanski P (2007). Volume, neuron density and total neuron number in five subcortical regions in schizophrenia. Brain.

[64] Pogarell O (2012). Dopaminergic neurotransmission in patients with schizophrenia in relation to positive and negative symptoms. Pharmacopsychiatry.

[65] Horn D, Ruppin E (1995). Compensatory mechanisms in an attractor neural network model of Schizophrenia. Neural Comput.

[66] Kim Y., Suh B.-C. Editorial: Brain cells’ compensatory mechanisms in response to disease risk factors. *Front Mol. Neurosci*. 15 10.3389/fnmol.2022.1096287 (2022). 10.3389/fnmol.2022.1096287 PMC9808396 36606142

[67] Ashraf A, Fan Z, Brooks DJ, Edison P (2015). Cortical hypermetabolism in MCI subjects: a compensatory mechanism?. Eur. J. Nucl. Med. Mol. Imaging.

[68] Blesa J (2017). Compensatory mechanisms in Parkinson’s disease: Circuits adaptations and role in disease modification. Exp. Neurol..

[69] Bhembre N, Bonthron C, Opazo P (2023). Synaptic compensatory plasticity in Alzheimer’s disease. J. Neurosci..

[70] Chen J. et al. Genetic relationship between Alzheimer’s disease and Schizophrenia. *Alzheimer’s Dement*. 18 10.1002/alz.065861 (2022).

[71] Dienel SJ, Lewis DA (2019). Alterations in cortical interneurons and cognitive function in schizophrenia. Neurobiol. Dis..

[72] Chokhawala K., Stevens L. *Antipsychotic Medications*. StatPearls [Internet]. Treasure Island (FL): StatPearls Publishing; https://www.ncbi.nlm.nih.gov/books/NBK519503/ (2023). 30137788

[73] Dazzan P (2005). Different effects of typical and atypical antipsychotics on grey matter in first episode psychosis: the ÆSOP study. Neuropsychopharmacology.

[74] Daskalakis ZJ (2002). Evidence for impaired cortical inhibition in Schizophrenia using transcranial magnetic stimulation. Arch. Gen. Psychiatry.

[75] Pittman-Polletta BR, Kocsis B, Vijayan S, Whittington MA, Kopell NJ (2015). Brain rhythms connect impaired inhibition to altered cognition in schizophrenia. Biol. Psychiatry.

[76] Anderson EJ (2017). Visual population receptive fields in people with Schizophrenia have reduced inhibitory surrounds. J. Neurosci..

[77] Cannon TD (2015). How Schizophrenia develops: Cognitive and brain mechanisms underlying onset of psychosis. Trends Cogn. Sci..

[78] Gonzalez-Burgos G, Lewis DA (2012). NMDA receptor hypofunction, parvalbumin-positive neurons, and cortical gamma oscillations in schizophrenia. Schizophr. Bull..

[79] Adams RA (2022). Computational modeling of electroencephalography and functional magnetic resonance imaging paradigms indicates a consistent loss of pyramidal cell synaptic gain in Schizophrenia. Biol. Psychiatry.

[80] Chen Y, Norton D, Ongur D (2008). Altered center-surround motion inhibition in schizophrenia. Biol. Psychiatry.

[81] Webler RD (2020). Decreased interhemispheric connectivity and increased cortical excitability in unmedicated schizophrenia: A prefrontal interleaved TMS fMRI study. Brain Stimul..

[82] Shin H-W, Chung SJ (2012). Drug-induced parkinsonism. J. Clin. Neurol..

[83] Hutton JT, Morris JL, Elias JW (1993). Levodopa improves spatial contrast sensitivity in Parkinson’s disease. Arch. Neurol..

[84] Ming W, Palidis DJ, Spering M, McKeown MJ (2016). Visual contrast sensitivity in early-stage Parkinson’s disease. Investig. Opthalmology Vis. Sci..

[85] Souza BOF, Abou Rjeili M, Quintana C, Beaulieu JM, Casanova C (2018). Spatial frequency selectivity is impaired in dopamine D2 receptor knockout mice. Front Integr. Neurosci..

[86] Takeshita S, Ogura C (1994). Effect of the dopamine D2 antagonist sulpiride on event-related potentials and its relation to the law of initial value. Int. J. Psychophysiol..

[87] Antal A, Kéri S, Bodis-Wollner I (1997). Dopamine D2 receptor blockade alters the primary and cognitive components of visual evoked potentials in the monkey, Macaca fascicularis. Neurosci. Lett..

[88] Afef O, Rudy L, Stéphane M (2022). Ketamine promotes adaption-induced orientation plasticity and vigorous network changes. Brain Res.

[89] Mehta MA, Manes FF, Magnolfi G, Sahakian BJ, Robbins TW (2004). Impaired set-shifting and dissociable effects on tests of spatial working memory following the dopamine D2 receptor antagonist sulpiride in human volunteers. Psychopharmacol. (Berl.).

[90] Puig MV, Miller EK (2015). Neural substrates of dopamine D2 receptor modulated executive functions in the Monkey prefrontal cortex. Cereb. Cortex.

[91] Martínez A (2008). Magnocellular pathway impairment in Schizophrenia: Evidence from functional magnetic resonance imaging. J. Neurosci..

[92] Martinez A (2012). Consequences of magnocellular dysfunction on processing attended information in Schizophrenia. Cereb. Cortex.

[93] Kim D, Wylie G, Pasternak R, Butler PD, Javitt DC (2006). Magnocellular contributions to impaired motion processing in schizophrenia. Schizophr. Res.

[94] Merigan WH, Maunsell JHR (1993). How parallel are the primate visual pathways?. Annu Rev. Neurosci..

[95] Amoruso L, Finisguerra A, Urgesi C (2020). Spatial frequency tuning of motor responses reveals differential contribution of dorsal and ventral systems to action comprehension. Proc. Natl. Acad. Sci..

[96] Braus DF, Weber-Fahr W, Tost H, Ruf M, Henn FA (2002). Sensory information processing in neuroleptic-naive first-episode Schizophrenic patients. Arch. Gen. Psychiatry.

[97] Gracitelli CP, Vaz de Lima FB, Bressan RA, Paranhos Junior A (2013). Visual field loss in schizophrenia: evaluation of magnocellular pathway dysfunction in schizophrenic patients and their parents. Clin. Ophthalmol..

[98] Zilles K, Palomero-Gallagher N (2017). Multiple transmitter receptors in regions and layers of the human cerebral cortex. Front Neuroanat..

[99] Beck K (2021). N-methyl-D-aspartate receptor availability in first-episode psychosis: A PET-MR brain imaging study. Transl. Psychiatry.

[100] Pilowsky LS (2005). First in vivo evidence of an NMDA receptor deficit in medication-free schizophrenic patients. Mol. Psychiatry.

[101] Michelson NJ, Kozai TDY (2018). Isoflurane and ketamine differentially influence spontaneous and evoked laminar electrophysiology in mouse V1. J. Neurophysiol..

[102] Ouelhazi A (2019). Effects of ketamine on orientation selectivity and variability of neuronal responses in primary visual cortex. Brain Res..

[103] Manookin MB, Weick M, Stafford BK, Demb JB (2010). NMDA receptor contributions to visual contrast coding. Neuron.

[104] Murray JD, Anticevic A (2017). Toward understanding thalamocortical dysfunction in schizophrenia through computational models of neural circuit dynamics. Schizophr. Res..

[105] Self MW, Kooijmans RN, Supèr H, Lamme VA, Roelfsema PR (2012). Different glutamate receptors convey feedforward and recurrent processing in macaque V1. Proc. Natl. Acad. Sci. USA.

[106] Moghaddam B, Javitt D (2012). From revolution to evolution: The glutamate hypothesis of schizophrenia and its implication for treatment. Neuropsychopharmacology.

[107] Zhang X, Sun Y, Liu W, Zhang Z, Wu B (2020). Twin mechanisms: Rapid scene recognition involves both feedforward and feedback processing. Acta Psychol. (Amst.).

